# Exploring a QoS Driven Scheduling Approach for Peer-to-Peer Live Streaming Systems with Network Coding

**DOI:** 10.1155/2014/513861

**Published:** 2014-07-10

**Authors:** Laizhong Cui, Nan Lu, Fu Chen

**Affiliations:** ^1^College of Computer Science and Software Engineering, Shenzhen University, Shenzhen 518060, China; ^2^Department of Computer Science and Technology, Beijing Foreign Studies University, Beijing 100089, China

## Abstract

Most large-scale peer-to-peer (P2P) live streaming systems use mesh to organize peers and leverage pull scheduling to transmit packets for providing robustness in dynamic environment. The pull scheduling brings large packet delay. Network coding makes the push scheduling feasible in mesh P2P live streaming and improves the efficiency. However, it may also introduce some extra delays and coding computational overhead. To improve the packet delay, streaming quality, and coding overhead, in this paper are as follows. we propose a QoS driven push scheduling approach. The main contributions of this paper are: (i) We introduce a new network coding method to increase the content diversity and reduce the complexity of scheduling; (ii) we formulate the push scheduling as an optimization problem and transform it to a min-cost flow problem for solving it in polynomial time; (iii) we propose a push scheduling algorithm to reduce the coding overhead and do extensive experiments to validate the effectiveness of our approach. Compared with previous approaches, the simulation results demonstrate that *packet delay*, *continuity index,* and *coding ratio* of our system can be significantly improved, especially in dynamic environments.

## 1. Introduction

P2P has become a dominant solution for distributing live video content to large populations of users in recent years, by leveraging clients' resources to serve each other. To provide the robustness and meet the streaming bandwidth requirement, most existing large-scale P2P live streaming systems organize peers into mesh. However, the streaming quality of them is not so satisfactory, especially in dynamic environments [1]. The performance bottleneck is due to the lack of a proper and optimal scheduling design.

Given a number of neighbors, a peer needs to decide which packets are transmitted by which neighbors, which is called scheduling. Existing scheduling can be broadly divided into two categories: pull and push. In the pull scheduling [2], the streaming is divided into blocks and a data structure called buffer map is periodically exchanged to reflect a peer which has blocks in its cache. According to the received buffer maps, each peer explicitly requests the desired blocks from its neighbors using some pull strategies, such as rarest-first or sequence-first. However, the* packet delay* is very large for this receiver-driven delivery and the bandwidth is not fully utilized since each block is only served by a peer at a time. To reduce the* packet delay*, the tree push scheduling [3] is introduced. Nevertheless, this strategy is not suitable for large-scale P2P live streaming. The reason lies in the following aspects: (1) the tree structure has large maintenance and repair costs in dynamic P2P environment; (2) since the leaf peers of the tree will not deliver content to any other peers, the bandwidth of the leaf peers is wasted. In the mesh push scheduling [4], the video streaming is divided into some substreams, and each peer reassembles all the substreams through receiving packets pushed by different neighbors. This method cannot solve the problems about performance degradation when the intensive system dynamic happens.

Network coding has been shown to be an effective way to improve the performance of P2P streaming by maximizing the network throughput and making the push scheduling feasible in mesh P2P [5]. Moreover, a missing segment of a peer can be served by multiple neighbors simultaneously. Mea and Baochun proposed a random push scheduling for network coding based P2P live streaming system called *R* ^2^ [6]. Through reducing the complexity of coordinated scheduling and improving the bandwidth resources, *R* ^2^ improves the system performance. However, they do not optimize the QoS metrics of transmission performance and the streaming quality so that they do not reach the optimal solution. Moreover, in *R* ^2^, before pushing a block, a peer has to produce a new coding block, which brings a lot of coding overhead since the operation of encoding consumes the computational resources. To generate new coding blocks by reencoding, a peer must receive enough coding blocks, which increases the extra packet delay. Since current scheduling cannot fully take advantages of network coding, it is necessary to redesign a QoS driven push scheduling approach for network coding based P2P live streaming system.

In this paper, we propose a novel QoS driven scheduling approach for network coding based P2P live streaming system with the following contributions. First, we introduce a new coding method, through combing the substreams with network coding. Second, we formulate the scheduling optimization problem and transform it to an equivalent min-cost flow problem for solving it in polynomial time. Third, we design a simple yet effective push scheduling algorithm to reduce the coding overhead and improve the robustness in dynamic environments. To evaluate the performance and effectiveness, we implement our approach on an event-driven P2P streaming simulator [7] and compare it with CoolStreaming [2] and *R* ^2^ [6]. The simulation results show that our approach improves the transmission performance and streaming quality by reducing the* packet delay* and improving the* continuity index*. Meanwhile, the coding overhead is lower by reducing the* coding ratio*, which reflects the better robustness of our approach in dynamic environments.

The rest of the paper is organized as follows. In Section 2, we discuss the related work. In Section 3, we present the details of our analysis model and introduce a new coding method. We propose the optimization and algorithm of our scheduling approach in Section 4. The simulation results are discussed in Section 5. Finally, Section 6 concludes our work.

## 2. Related Work

The tree-based P2P live streaming systems, such as [3, 8, 9], could reduce the playback delay. In the tree topology, the root is the streaming servers and all other peers are organized into one or more multicast trees. The streaming content of the server is decomposed into substreams that are pushed through corresponding trees from the server to all nodes in that tree. Although such tree-based push scheduling algorithm is beneficial in reducing the delays of transmitting data, they are not suitable to deploy in real-world large-scale streaming systems. The main reason is its complexity and cost involved in maintaining the tree topology in dynamic P2P environment.

CoolStreaming [2] is a mesh based P2P live streaming system and it can serve large-scale users. In the mesh based systems, the streaming content is divided into a series of segments and each represents a short duration content of playback. A new concept, called buffer map, is introduced to represent the segment available information of each peer. To know which segments each neighbor has, the buffer map is periodically exchanged among peers. CoolStreaming proposes a rarest-first pull scheduling algorithm, which means the rarest segment among its neighbors is transmitted preferentially. Although the systems with the mesh topology and pull scheduling algorithm are more robust to peers dynamic than the systems with the tree topology and push scheduling algorithm, they inevitably increase the delay of data transmission from servers to all participating peers. These delays mainly come from the periodic exchange of buffer map and explicit segment request. Zhang et al. [4] propose Grid Media to improve the delay of CoolStreaming, which is also a mesh based P2P live streaming system. It combines a hybrid scheduling algorithm, consisting of the pull and push modes. In Grid Media, a peer requests the streaming packets with the pull mode at startup and then relays streaming packets in the push mode. Essentially, to utilize the push mode, the streaming content is divided into multiple substreams, each of which is pushed in a different tree structure. However, it also needs to exchange the segment available information among peers and is not robust due to the dynamic environment.

For dealing with the defects of mesh based P2P streaming system, some coding methods are introduced into P2P streaming system, the most representatives of which are rateless codes and network coding. The rateless fountain codes, including LT codes [10], Raptor codes [11], and online codes [12], can be readily used in peer-to-peer streaming with substantial advantage. The typical P2P system with rateless codes is rStream. In rStream [13], the Raptor codes are used in P2P streaming system to eliminate the coordination of the content available information. The peer selection and rate allocation are formulated as an optimization problem and the algorithm is also proposed to solve the optimization problem. Although it improves the end-to-end latency, it neglects the other QoS metrics, such as throughput and redundancy.

Recently, network coding [14–16] has been widely used to improve the performance of P2P systems. Gkantsidis and Rodriguez [17, 18] have proposed that randomized network coding can significantly reduce the downloading times in P2P content distribution and file downloading systems. Lava [5] fairly evaluates the feasibility and effectiveness of random network coding [19] for P2P live streaming systems. While Lava has focused on a fair comparison study without improving the P2P live streaming traditional mechanism, the advantages of network coding have not been fully explored. Inspired by Lava, Mea and Baochun [6] redesign the scheduling algorithm and propose a random push with random network coding scheduling algorithm called *R* ^2^ to take full advantage of network coding. The random push scheduling algorithm of *R* ^2^ is revised to be suitable for UUSee [20], which is a popular P2P VoD system. It demonstrates that network coding with random push scheduling algorithm can also improve the P2P VoD system. Sarkar and Wang [21] give the details of the setup through a measurement study of network coding in real P2P VoD system. Sarkar and Wang [22] propose a prefetch strategy for network coding based P2P VoD systems. Nguyen and Nakazato [23] discuss that the rare-first scheduling algorithm is not enough for P2P streaming with network coding. Sheikh et al. [24] propose a distributed media-aware scheduling algorithm for P2P streaming with network coding, which considers the feedback information of neighbors, including loss rate and decoding ratio.

Although the network coding in P2P streaming is effective and practical, the research of the scheduling algorithm in network coding based P2P streaming systems is still an open research area, especially in optimizing several QoS metrics. Hsu [25] produces a knowledge sharing method and Mishra and Srivastava [26] discuss the information spreading behavior in the distributed systems, both of which enlighten our work indirectly. Our idea of QoS driven scheduling algorithm is partly inspired by the previous research, but we introduce a new coding method, formulate an optimization for the scheduling problem, and propose the corresponding distributed solution.

## 3. The Analysis Model and Coding Method

We let *R* bits/s be the streaming rate of the live stream. To realize the push scheduling in mesh [4], we also divide the live stream into several substreams. Let each substream's rate be *γ*. It means the live stream is divided into *N* substreams, *N* = *R*/*γ* (assuming that *R* is divisible by *γ*). On the other hand, as for the traditional P2P live streaming with network coding, the live stream is divided into several segments, and each segment is divided into several blocks. The network coding is only used in each segment to generate coding blocks, without encoding blocks across different segments.

In our approach, we propose a network coding method by combining the substream with network coding, called coding substream. The details of the coding method of coding substream are described as follows. We let the live stream be divided into segments. Each segment has a sequence number called *segment*_*id* and is divided into *M* blocks further. The network coding is used in each segment to generate *M*′ coding blocks. We directly utilize random network coding and progressive decoding method [5]. Whenever a peer wants to encode a block, it first independently and randomly chooses a set of coding coefficients [*e*_1_, *e*_2_,…, *e*_*m*_] in the Galois field GF(2^8^) and then produces a coding block *x*, using the following equation:

(1) $$\begin{array}{c} x=\overset{M}{\underset{i=1}{\sum }}e_{i}\cdot b_{i}. \end{array}$$

When a peer receives *M* linearly independent coding blocks *x* = [*x*_1_, *x*_2_,…, *x*_*M*_], it can decode the original segment as follows. It extracts the coefficients of each encoded block *x*_*i*_ to form the *M* × *M* coefficient matrix *E*. Then, it recovers the original segment *b* = [*b*_1_, *b*_2_,…, *b*_*M*_] as (2). We utilize Gaussian elimination [5] to solve this equation. Consider the following:

(2) $$\begin{array}{c} b=E^{-1}x^{T}. \end{array}$$

**Figure 1:**
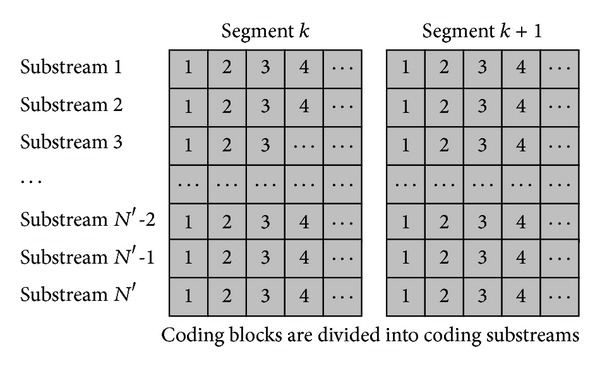
The coding method and the coding substreams.

The *M*′ coding blocks are interleaved into *N*′ coding substreams, *N*′ = ⌊*B*_*s*_/*γ*⌋, *M*′ = *M*∗(*N*′/*N*) (*B*_*s*_ is the upload bandwidth of source server). Each substream has a sequence number called *sub*-*stream*_*id*. Each coding block in one coding substream has a sequence number called *block*_*id*. Thus, each coding block is identified by a triple, <*segment*_*id*, *sub* − *stream*_*id*, *block*_*id*>. A diagram of this coding method and coding substreams is shown in Figure 1.

We take an example to explain this method. Assume that *N* = 3, *M* = 6, and *B*_*s*_ = 2*R*, which means the original segment includes 6 blocks and each segment is divided into 3 substreams; namely, each substream has 2 blocks. To generate the coding substreams, it will produce *M*′ = 2*M* = 12 coding blocks (with the *block*_*id* 1,2,3,11,12), for a segment and these 12 coding blocks are divided into *N*′ = 2*N* = 6 coding substreams, that is, coding substreams 1 with coding blocks {1,7}, coding substreams 2 with coding blocks {2,8},…, and coding substreams 6 with coding blocks {6,12}.

In our approach, the network coding operation is also employed both on the source server side and on the peers side. However, the encoding operations mainly happen on the server and occasionally happen on the peers when necessary (this situation will be discussed later), which is different from the traditional network coding based P2P streaming systems, such as Lava [5] and *R* ^2^ [6]. This design could effectively reduce the coding overhead.

In the traditional push scheduling and non-network coding systems [4], each peer needs to collect all *N* substreams for smooth playback. This brings the limitation that the peers may fail to get enough substreams since the system is highly dynamic. However, in our approach, we apply the network coding to the *M* original blocks of a segment for producing *M*′ coding blocks with little probability of duplication, which also means that it can produce more available substreams, that is, from *N* substreams to *N*′ substreams. On the one hand, our approach increases the diversity of the content and robustness of the system. On the other hand, it decreases the complexity of the substreams scheduling. Each peer could subscribe to any *N* substreams from these *N*′ substreams. As long as the peer collects *M* linearly independent coding blocks from *N* substreams, it can decode the original content.

**Figure 2:**
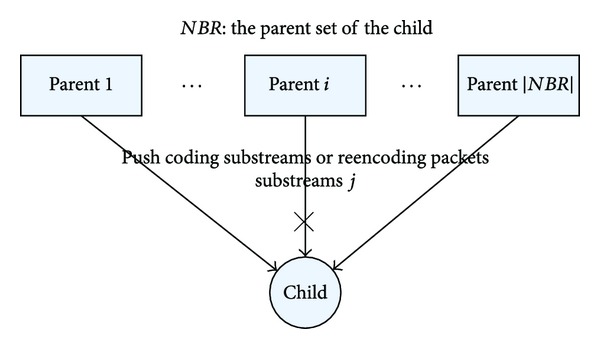
The scheduling process of a child and its parents.

We formulate this simple coding substreams scheduling as the following model. In the transmission process based on coding substreams, each peer subscribes to *N* coding substreams via its neighbors. Once a peer receives coding blocks from its subscribed substreams, it also relays the content to its downstream neighbors that requested the corresponding substreams. We can decompose the transmission process of the whole system into several transmission units. A typical transmission unit is illustrated in Figure 2, in which the focused peer is called child and the peers serving substreams are called parents. Without loss of generality, we can solve the substreams scheduling problem by focusing on a certain child and its parents in Figure 2. The child has a set of parents denoted by *NBR*. For each parent *i* in *NBR*, it will allocate a certain upload bandwidth *B*_*i*_ bits/s to the child for the coding substreams transmission. Let *h*(*i*, *j*)∈{0,1} denote whether parent *i* could provide substream *j*. *h*(*i*, *j*) has value 1 if parent *i* could provide the substreams *j* and 0 otherwise. When a child first joins the system, it requests a neighbor list as its parent set *NBR* from the bootstrap server. Actually, any neighbor selection algorithm can be used in our approach. After obtaining the parent set, the child asks each parent for their buffer information about the coding substreams (i.e., the vector [*h*(*i*, *j*)] = (*h*(*i*, 1), *h*(*i*, 2),…, *h*(*i*, *N*′))). Upon receiving each parent's vector [*h*(*i*, *j*)] = (*h*(*i*, 1), *h*(*i*, 2),…, *h*(*i*, *N*′)), the child solves the scheduling problem about arranging the coding substreams that means it needs to decide to subscribe to which coding substream via which parent for obtaining the *N* coding substreams.

## 4. The Optimization and Algorithm for the Scheduling Problem

We first formulate the scheduling problem as a cost optimization problem and introduce a polynomial time solution in Section 4.1. And then, we give the details of the push scheduling algorithm of our approach in Section 4.2.

### 4.1. The Optimization of the Scheduling Problem

Let *o*_*i*_ be the maximal substreams which can be pushed to the child from parent *i*, *i* ∈ *NBR*. It is calculated by (3), in which *B*_*i*_ is the upload bandwidth that parent *i* allocated to the child and *γ* is the streaming rate of each coding substream. Consider

(3) $$\begin{array}{c} o_{i}=\left\lfloor \frac{B_{i}}{\gamma }\right\rfloor . \end{array}$$

Before formulating the optimal scheduling problem, we define a cost function *C*(*i*, *j*) as (4), which represents the average transmission delay of a packet considering the packet loss probability when the substream *j* is assigned to parent *i*. The larger *C*(*i*, *j*) means higher transmission delay. *ρ*_*i*,*j*_ represents the child's packet loss probability of coding substream *j* from parent *i*. *D*_*i*,*j*_ denotes the child's link latency of substream *j* from parent *i*. We use an example to explain our design of the cost function *C*(*i*, *j*). For two parents *i* and *k*, we assume that *D*_*i*,*j*_ = 3 seconds, *D*_*k*,*j*_ = 1 second, *ρ*_*i*,*j*_ = 0.1, *ρ*_*k*,*j*_ = 0.3, and 10 packets are transmitted in each sending period. For parent *i*, the child receives 9 packets in 3 seconds, namely, using 1/3 second per packet, and for parent *k*, the child receives 7 packets in 1 second, namely, using 1/7 second per packet. Apparently, the child should prefer to subscribe to coding substream *j* via parent *k*. Consider the following:

(4) $$\begin{array}{c} C\left(i,j\right)=D_{i,j}\times \frac{1}{\rho_{i,j}}. \end{array}$$

Given the coding substreams vector [*h*(*i*, *j*)] = (*h*(*i*, 1), *h*(*i*, 2),…, *h*(*i*, *N*′)) from parent *i* and the maximal substreams allocated to the child *o*_*i*_, ∀*i* ∈ *NBR*, the goal of the scheduling problem is to find a solution of subscribing to which coding substream via which parent, achieving the minimum total transmission cost of the *N* subscribed coding substreams for the child. We formulate it as the optimization scheduling problem with some given restrictions in the following equation:

(5) Minimize  ∑j=1N′ ∑i∈NBRxijh(i,j)C(i,j)subject  to  (a) x(i,j)∈0,1, i∈NBR,j∈{1,2,…,N′}(b)∑i∈NBRx(i,j)=1, ∀j(c)∑j=1N′x(i,j)≤oi,(d)∑j=1N′ ∑i∈NBRxij=N.

From constraint (a), *x*_*ij*_ is a decision variable, which has value 1 if the substream *j* is assigned to parents *i* and 0 otherwise. It indicates this scheduling optimization is a 0-1 programming problem. The constraint (b) means the child can only subscribe to one coding substream via one parent. The constraint (c) means parent *i*'s number of subscribed coding substreams must be smaller than its upper bound *o*_*i*_. The constraint (d) means the child only needs *N* coding substreams.

The classical min-cost flow problem could be formulated as (6). The min-cost flow problem is a well-known optimization problem. Since the min-cost flow problem is a convex problem, which could be used by several algorithms to get the solution in polynomial time. Consider

(6)   min⁡  ∑(i,j)∈Ac(i,j)x(i,j)subject  to  (a)∑j:(i,j)∈Ax(i,j)−∑j:(j,i)∈Ax(j,i)=di,∀i∈V(b)0≤x(i,j)≤u(i,j), ∀(i,j)∈A.

For solving this optimization problem in polynomial time, we propose some transformation rules to transform this scheduling optimization problem to an equivalent min-cost flow problem. The transformation rules are described in Table 1, which includes two aspects: vertexes and edges. The key idea of our transformation rules lies in two aspects: (1) we use two type vertexes to represent the parents and coding substreams; (2) besides the edges between the vertex *p*_*i*_ and vertex *ss*_*j*_, the cost of other edges is 0.

**Figure 3:**
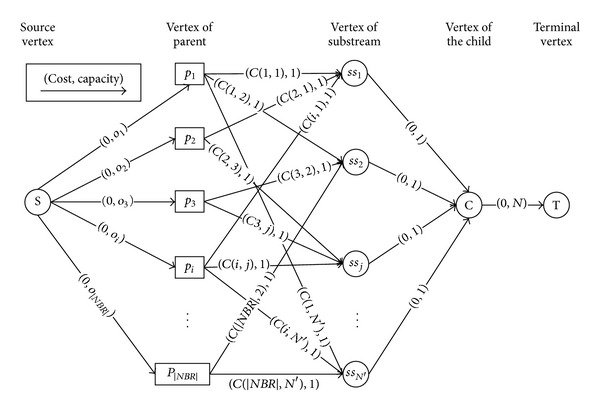
A min-cost-transformation example.

Figure 3 shows the transform result. We use the double scaling algorithm [27] to solve this min-cost flow problem in polynomial time. In the optimal solution, the flow amount on edges (*p*_*i*_, *ss*_*j*_) is the value of *x*(*i*, *j*). With the optimal solution, we can also get the scheduling decision of the child; that is, for each *x*(*i*, *j*) = 1, the substream *j* is assigned to parent *i*.

### 4.2. Push Scheduling Algorithm

We also design a push scheduling algorithm to ensure the coding blocks transmission of substreams with low delay and overhead. As traditional P2P live streaming, each peer has a buffer with a limited constant length. When a child receives a coding block from the subscribed coding substream, it puts this block into its buffer and does one step progressive decoding operation by applying Gauss-Jordan elimination [5], which can reduce the decoding time. For serving the child, a parent has two modes to push its content: Forwarding Push and Reencoding Push. According to the status of child's buffer, the parent makes a choice from these two modes.

#### 4.2.1. Forwarding Push

The parent directly forwards the coding block in its buffer to the child only if the child lacks the block in the corresponding and subscribed substreams. When a child subscribes to a coding substream via a parent, it should inform the largest *block*_*id* of the block it has received of each segment in that substream. If the parent has some blocks, the *block*_*id* of which is larger than its child's largest *block*_*id*, it can directly forward them to its child and at the same time it updates the child's largest *block*_*id*.

#### 4.2.2. Reencoding Push

If the parent cannot find any block in its buffer that can be directly forwarded to the child (it means that the child has received the whole coding blocks of its subscribed substreams from this parent), it will try to produce a new coding block through reencoding the received coding blocks in its buffer and push the new coding block to the child. To ensure the new produced coding block is linearly independent of the child, we import the concept of* coding aggressiveness α*(0 < *α* < 1) [5]. A parent can produce a new coding block by re-encoding the received coding blocks only if the percentage of received coding blocks in that segment is larger than *α* (the segment is called segment exists). After producing a new coding block by re-encoding, the parent will push it to the child. Upon receiving this block, the child will insert it into a missing substream and assign it with the largest *block*_*id* in that substream.

This design of Reencoding Push makes our system more robust in dynamic environment. We explain it through an example in Figure 2. Assume the child subscribes to coding substream *j* via parent *i* and the parent *i* suddenly leaves the system. At this time, the child needs to search for another parent by contacting the bootstrap server or any other membership mechanisms. Anyway, this parent repair process will take some time. During this period, the child may not receive the content in time and have to suffer from the incomplete streaming that is decreasing the streaming quality. However, with Reencoding Push, the child's other parents will discover coding substream *j* is missing. Although the child does not subscribe to the coding substream *j* via any parent of them, they will reencode to produce new coding blocks, in order to supply the missing substream *j*. After receiving enough reencoding blocks, the child could decode the original content and provide smooth playback. Besides, in traditional push-mesh system, the child has to find a new parent which can provide the exact same substream *j*. In our approach, the child need not find the exact same coding substream *j*, and it needs only to find a coding substream *j*′, which has not been subscribed before. Since the coding substream *j*′ is linear independence with the remaining *N* − 1 coding substreams of the child, it can replace substream *j* to be used for decoding and therefore the parent repairing process could be shortened effectively.

**Figure 4:**
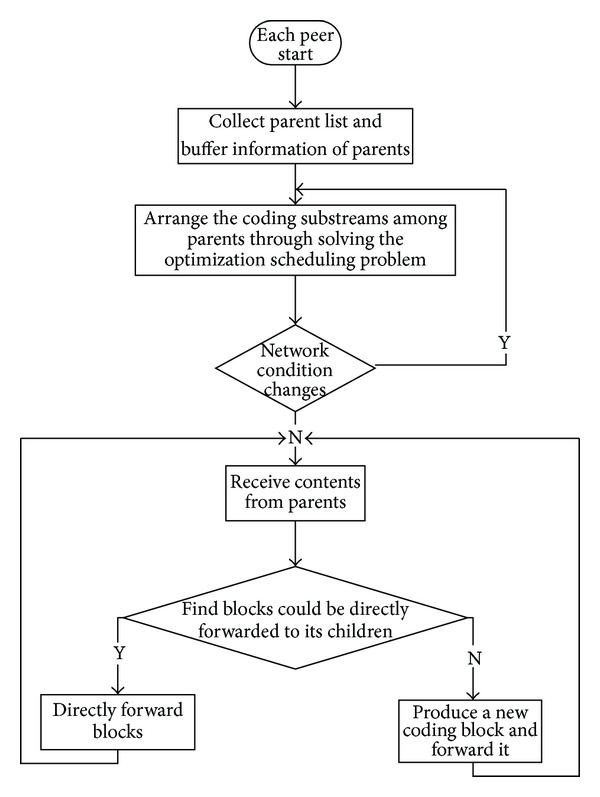
The flowchart of our QoS driven scheduling approach.

We summarize our QoS driven scheduling approach by describing the flowchart as in Figure 4. In the beginning of a new child joining the system, it contacts the server to get the neighbor list as its parent set. Then it asks for parents' buffer information (i.e., the vector [*h*_*i*,*j*_]'s). Upon receiving the vector *h*_*i*,*j*_ from all parents, the child computes a new scheduling for these substreams, through solving the optimization as (5). The child recomputes the scheduling when it meets large changes about network conditions, such as the departure of parents or congestion in a certain connection. After deciding the substreams subscription, the child will receive content from parents and push the content to its child via two modes of our push scheduling algorithm.

## 5. Evaluation

### 5.1. Simulation Setup and Metrics

We utilize a discrete event-driven packet level simulator [7] and realize the network coding operation and our scheduling approach on it. We conduct a series of extensive simulations to study the impacts of our scheduling approach. For comparison, we simulate two conventional systems: the classic system CoolStreaming [2] with traditional pull and state-of-the-art network coding based system *R* ^2^ [6] with random push.

In our simulation, all streaming and control packets including the sending and receiving buffer of each peer are carefully simulated. In all experiments, unless specified otherwise, we set the original streaming rate *R* = 400 Kbit/s and the substream rate *γ* = 50 Kbit/s. Each segment represents 1 second of the playback, which means each segment is 400 Kbit/s long. The segments are divided into *M* = 320 blocks. According to literature [5], the* coding aggressiveness α* = 0.5. Each peer has 15 parents. The upload capacity of the source server is *B*_*s*_ = 5 Mbit/s, which is a reasonable ratio in practice. The default number of peers is 200. The default parameters of the simulated system are set as Table 2. We employ real-world end-to-end latency matrix (2500 × 2500) measured on Internet [28] and the transmission loss rate for data packets between two peers as uniformly distributed from 0.02 to 0.1, which are typical in Internet. To simulate the bandwidth heterogeneity of peers, we define three different typical ADSL peers. The default of the peer bandwidth distribution is presented in Table 3, which is measured on Internet [29].

Besides the simulation with default parameters values in Table 2, we also change the peer number and streaming rate to do extra experiments. As the values of the peer number and streaming rate change, the peer bandwidth distribution has to be reset, in order to meet the increase of system demand for peers bandwidth. The details of the corresponding peer bandwidth distribution are described in Table 4.

We focus on the following metrics in our evaluation.

*Packet Delay*. It refers to the delay between the time when the packet is sent out from the source server and when it is received at a peer after several hops. 

*Continuity Index*. It is defined as the fraction of the segments that could be received and decoded before their playback deadlines. 

*Coding Ratio*. It is defined as the percentage of the transmitted blocks produced by encoding operation over all the transmitted blocks that are sent by peers.

### 5.2. Simulation Results

#### 5.2.1. *Packet Delay*

**Figure 5:**
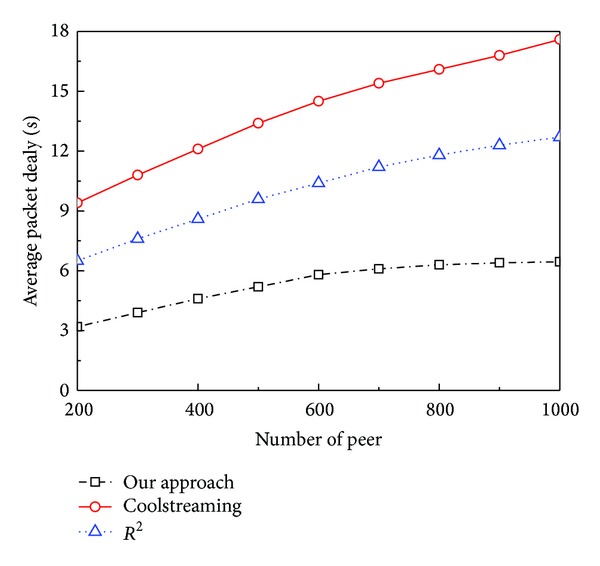
Average* packet delay* versus number of peers.

Figure 5 shows the average* packet delay* of the three systems versus the number of peers. Generally, the average* packet delay* increases with the number of peers, since the network scale of systems becomes large. The average* packet delay* of CoolStreaming is the largest, as the pull scheduling algorithm accumulates large* packet delay* along the transmission path. Since network coding makes the push scheduling feasible in mesh P2P systems, *R* ^2^ reduces* packet delay*. However, it uses random push scheduling without considering the link transmission delay and the frequent reencoding operations make the* coding ratio* too high (in Figure 9). So, its* packet delay* is still larger than ours. Our system achieves the smallest* packet delay*. The reasons are that our scheduling optimization chooses the parents with low link transmission delay to push coding substreams in a timely mode and our scheduling algorithm reduces the reencoding operations as much as possible.

**Figure 6:**
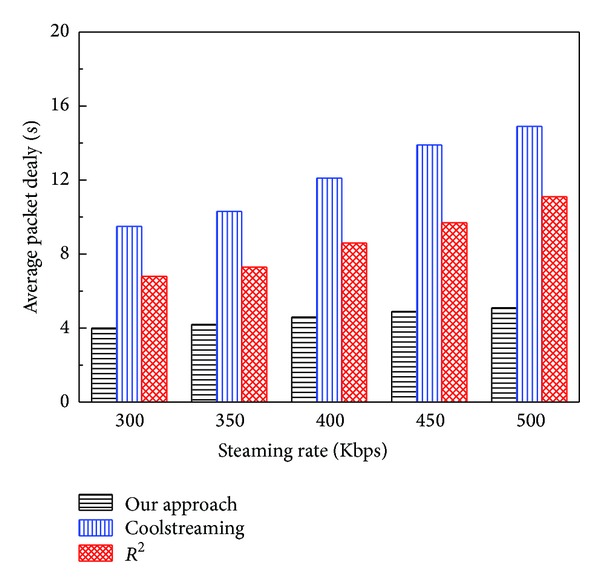
Average* packet delay* versus streaming rate.

The average* packet delay* of the three systems versus streaming rate is illustrated in Figure 6. In general, the average* packet delay* becomes larger with the increase of streaming rate, as the quantity of transmitted data increases. The reason is that with larger streaming rate, a segment has more blocks and it has to take more times to receive these blocks and decode them. Overall, as the streaming rate increases, although the packet delay inevitably increases, our approach can keep it at a more reasonable and lower level than those of CoolStreaming and *R* ^2^, which means our algorithm has better scalability.

#### 5.2.2. *Continuity Index*

**Figure 7:**
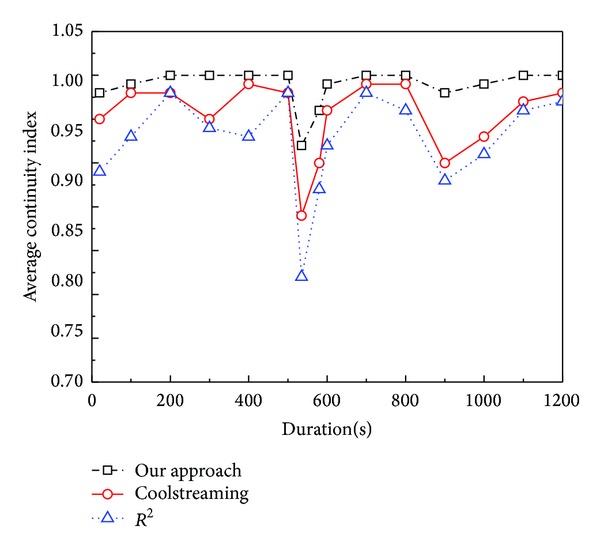
Average* continuity index* versus simulation duration.

The streaming quality is measured by* continuity index*.  Figure 7 shows the average* continuity index* of the three systems versus simulation duration. To simulate the dynamic environment, we let 50 peers and 20 peers leave the system, respectively, at 500 s and 800 s of the duration. Our system achieves the highest* continuity index*. The* continuity index* of ours has the least reduction, which means our systems have the best robustness in dynamic environment. The reason mainly lies in two aspects. (i) Our approach considers the packet loss probability and transmission delay in the optimization scheduling problem. This optimization solution ensures the packet delay can be reduced as much as possible; that is, most segments could be received and decoded before their playback deadlines. (ii) The design of Reencoding Push mode ensures the child can still receive enough blocks in dynamic environment.

**Figure 8:**
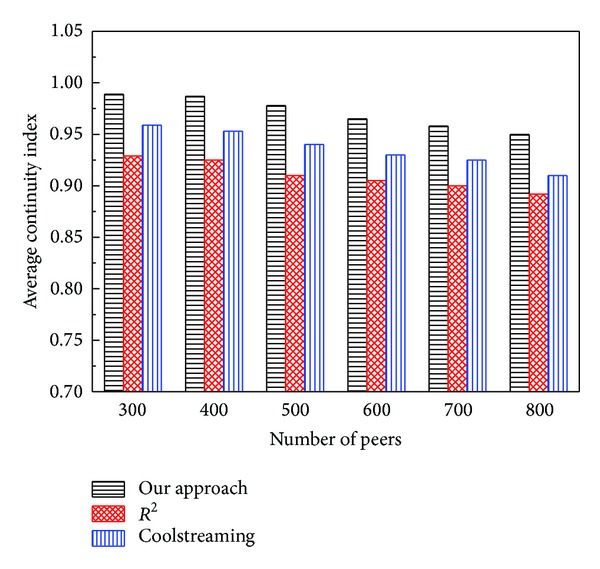
Average* continuity index* versus peer number.

The average* continuity index* of the three systems versus the number of peers is illustrated in Figure 8. In general, the average* continuity index* becomes smaller with the increase of system scale. However, the decrease amplitude of our* continuity index* is slight and the* continuity index* of our approach can still keep at a high level, which demonstrates that our approach can keep good scalability. The reason mainly lies in two aspects. (i) The* packet delay* of CoolStreaming and *R* ^2^ becomes larger (described in Figure 5), so that, as for CoolStreaming, some segments cannot arrive at the peers before the playback deadline of these segments, and as for *R* ^2^, the peers also cannot receive enough blocks to decode the segments in time. (ii) In our approach, the segments, not decoded and close to the playback deadline, still have chances to obtain the absent coding blocks through our Reencoding Push mode, which will increase the decoding probability of these segments. Our approach has improved CoolStreaming and *R* ^2^ in terms of these two aspects. Thus, more segments could be decoded before their playback deadline.

#### 5.2.3. *Coding Ratio*

**Figure 9:**
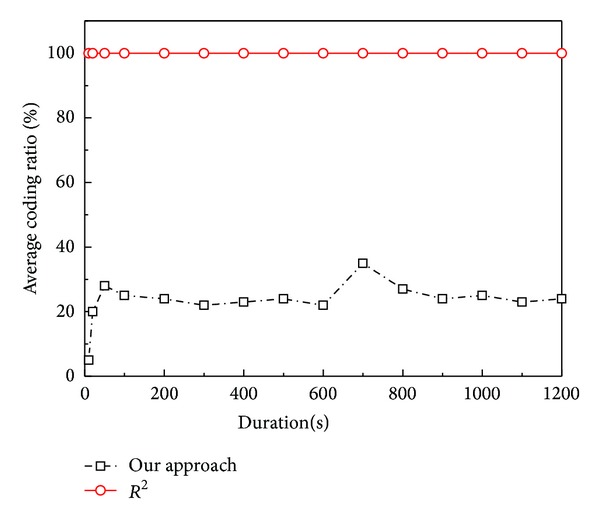
Average* coding ratio* versus simulation duration.

The encoding operation of network coding not only consumes the computing resources but also increases the packet transmission delay. Since encoding operations bring obvious coding overhead, it should be reduced as much as possible. We use* coding ratio* to measure the coding overhead. To simulate the dynamic environment, we let 20 peers leave the system at 600 s of the duration. Figure 9 shows the average* coding ratio* of the two systems, our system and *R* ^2^ versus simulation duration. For *R* ^2^, the* coding ratio* is nearly always 100% since each peer has to produce a new coding block by encoding operation before serving a packet to its neighbor. In our push algorithm, most coding blocks are directly forward to the child and a new coding block will be produced for the child by Reencoding Push only if necessary. Therefore, the* coding ratio* of our system is less than 30% most of the time. When some peers leave the system, the reencoding operations briefly increase to supply missing substreams in the parent repair process. So, our system achieves better overhead control and robustness in dynamic environment.

**Figure 10:**
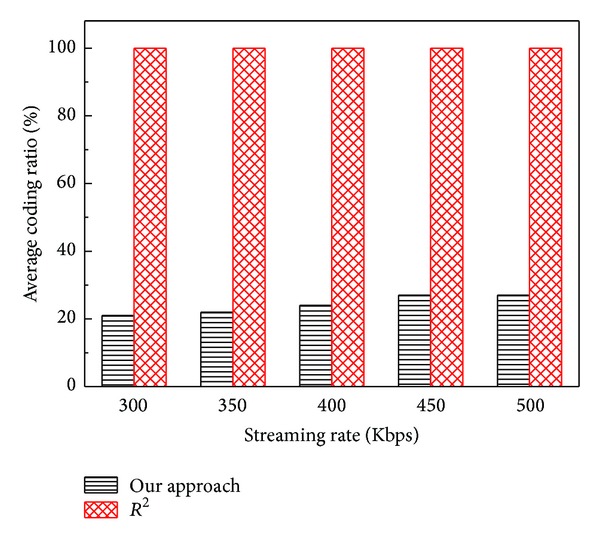
Average* coding ratio* versus streaming rate.

The average* coding ratio* of the two systems, our system and *R* ^2^, versus the streaming rate is illustrated in Figure 10. The average* coding ratio* of *R* ^2^ is also nearly always 100%. Whatever the streaming rate is, each peer of *R* ^2^ needs to generate a new coding block before transmitting a packet to any of its neighbors. The average* coding ratio* of our approach increases as the streaming rate becomes larger. The reason is that both the quantities of transmitted data and* packet delay* increase, so that the peers have to use longer time to obtain all the blocks and the quantity of loss packets becomes larger. This situation leads to the probability that the parent cannot forward any block in its buffer to its child will increase a little. According to our approach, the reencoding operations will increase slightly. The increase amplitude of our* coding ratio* is slight and the* coding ratio* of our algorithm can still keep at a low level, which demonstrates that our algorithm can keep good scalability.

## 6. Conclusions

In this paper, we study and propose a QoS driven scheduling approach for network coding based P2P live streaming system. Through introducing a new network coding method for substreams, we reduce the complexity of the scheduling problem, which is formulated as an optimization problem. Furthermore, we transform the optimization problem to an equivalent min-cost flow problem to solve it in polynomial time and propose a push scheduling algorithm to reduce the coding overhead. We conducted extensive simulation to validate the performance and effectiveness of our approach compared with other traditional and state-of-the-art schemes. Experimental results show that our approach achieves better transmission performance and streaming quality with substantially much lower overhead in dynamic environments.

## Figures and Tables

**Table 1:** Transformation rules.

Transformation rules of vertexes	
(1)	We add two virtual vertexes, source vertex *S* and terminal vertex *T*.
(2)	We add |*NBR*| parent vertexes, each of which is represented by *p*_*i*_, *i*∈*NBR*.
(3)	We add the vertex *ss*_*j*_ to express the substream *j*, *j*∈{1, 2,…, N′}.
(4)	We add the vertex *C* to express the child node, which makes the scheduling strategy.

Transformation rules of edges	

(5)	We add the edge between vertex *S* and vertex *p*_*i*_, the cost of which is 0 and capacity of which is *o*_*i*_.
(6)	For ∀*j*∈{1, 2,…, N′}, if *h*(*i*, *j*) = 1, we add the edge between the vertex *p*_*i*_ and vertex *ss*_*j*_, the cost of which is *C*(*i*, *j*) and the capacity of which is 1.
(7)	For ∀*j*∈{1, 2,…, N′}, we add the edge between the vertex *ss*_*j*_ and the vertex *C*, the cost of which is 0 and the capacity of which is 1.
(8)	We add the edge between vertex *C* and vertex *T*, the cost of which is 0 and capacity of which is *N*.

**Table 2:** The default parameters of the simulation.

Category	Parameter value
Peer number	200
Substream rate *γ*	50 Kbit/s
Streaming rate *R*	400 Kbit/s
Segment length	1 s (320 blocks)
Parent count	15
Upload bandwidth of streaming server *B*_*s*_	5 Mbit/s
Coding aggressiveness *α*	0.5

**Table 3:** Peer bandwidth distribution.

Category	Downlink	Uplink	Ratio (default)
A	768 Kbit/s	128 Kbit/s	30%
B	1.5 Mbit/s	384 Kbit/s	40%
C	3 Mbit/s	1 Mbit/s	30%

**Table 4:** Peer bandwidth distribution versus peers number and streaming rate.

The variation of peers number		The variation of streaming rate	
Peer number	Category ratio (A/B/C)	Streaming rate (Kbit/s)	Category ratio (A/B/C)
300	35%/45%/20%	300	40%/40%/20%
400	35%/40%/25%	350	35%/45%/20%
500	30%/45%/25%	400	30%/40%/30%
600	40%/30%/30%	450	30%/35%/35%
700	20%/35%/45%	500	20%/35%/45%
800	20%/30%/50%		
